# Warm-season temperatures and emergency department visits among children with health insurance

**DOI:** 10.1088/2752-5309/ac78fa

**Published:** 2022-11-01

**Authors:** Jennifer D Stowell, Yuantong Sun, Keith R Spangler, Chad W Milando, Aaron Bernstein, Kate R Weinberger, Shengzhi Sun, Gregory A Wellenius

**Affiliations:** 1 Department of Environmental Health, Boston University School of Public Health, Boston, MA, United States of America; 2 Boston Children’s Hospital, Boston, MA, United States of America; 3 School of Population and Public Health, University of British Columbia, Vancouver, British Columbia, Canada; 4 Optum Labs Visiting Scholar, Eden Prairie, MN, United States of America

**Keywords:** heat, children, climate change, morbidity

## Abstract

High ambient temperatures have become more likely due to climate change and are linked to higher rates of heat-related illness, respiratory and cardiovascular diseases, mental health disorders, and other diseases. To date, far fewer studies have examined the effects of high temperatures on children versus adults, and studies including children have seldom been conducted on a national scale. Compared to adults, children have behavioral and physiological differences that may give them differential heat vulnerability. We acquired medical claims data from a large database of commercially insured US children aged 0–17 from May to September (warm-season) 2016–2019. Daily maximum ambient temperature and daily mean relative humidity estimates were aggregated to the county level using the Parameter-elevation Relationships on Independent Slopes dataset, and extreme heat was defined as the 95th percentile of the county-specific daily maximum temperature distribution. Using a case-crossover design and temperature lags 0–5 days, we estimated the associations between extreme heat and cause-specific emergency department visits (ED) in children aged <18 years, using the median county-specific daily maximum temperature distribution as the reference. Approximately 1.2 million ED visits in children from 2489 US counties were available during the study period. The 95th percentile of warm-season temperatures ranged from 71 °F to 112 °F (21.7 °C to 44.4 °C). Comparing 95th to the 50th percentile, extreme heat was associated with higher rates of ED visits for heat-related illness; endocrine, nutritional and metabolic diseases; and otitis media and externa, but not for all-cause admissions. Subgroup analyses suggested differences by age, with extreme heat positively associated with heat-related illness for both the 6–12 year (odds ratio [OR]: 1.34, 95% confidence interval [CI]: 1.16, 1.56) and 13–17 year age groups (OR: 1.55, 95% CI: 1.37, 1.76). Among children with health insurance across the US, days of extreme heat were associated with higher rates of healthcare utilization. These results highlight the importance of individual and population-level actions to protect children and adolescents from extreme heat, particularly in the context of continued climate change.

**Abbreviations**
ED: emergency department event
OLDW: Optum Labs Data Warehouse
PRISM: parameter-elevation relationships on independent slopes

## 1. Introduction

The adverse health impacts of exposure to days of high ambient temperatures (i.e. ‘heat’) have been widely studied in adults. Heat has been linked to a higher risk of various adverse health outcomes, including death and/or healthcare utilization for heat-related illnesses, renal, cardiovascular, and respiratory diseases, and mental health conditions [1–13]. However, most of this research has been conducted among adults, with relatively less known regarding the impacts of heat on children [14–19]. There is evidence suggesting that, in the context of extreme heat, children may have different heat tolerance compared to adults [20]. Moreover, children’s perception of heat may differ from those of adults and, coupled with their dependence on caregivers, limit their ability to identify signs or symptoms related to excess heat exposure or protect themselves appropriately [21, 22]. Providing those who care for children with a better understanding of the potential adverse health impacts of heat may help protect children’s health during periods of extreme heat [23, 24].

A growing body of literature suggests that multiple pediatric conditions may be sensitive to heat, including heat-related illnesses, injuries, gastrointestinal illnesses, and general symptoms [25]. For example, Knowlton *et al* observed that rates of emergency department (ED) visits in children 0–4 years of age were 5% higher during a severe heatwave in California in 2006 versus a cooler reference period [26]. In New York City, Sheffield *et al* showed that, among children 0–4 years old, summertime temperatures were positively associated with higher rates of ED visits for all-cause and select specific causes [14]. In subsequent analyses using the same data source, Niu *et al* confirmed these findings in the youngest children (0–4 years) and showed similar associations in both older children (5–12 years) and adolescents (13–18 years) [27]. A large study across 18 cities in China showed that temperature was more strongly associated with ED visits in those aged <18 years versus older individuals, suggesting that children are a particularly susceptible subpopulation [4]. Studies in other locations have provided similar insights, albeit with some heterogeneity in results across studies [28–32]. Of note, very few studies have investigated this issue beyond a specific city, state, or region. One exception is a recent study that leveraged administrative claims for ED visits from 47 children’s hospitals across the US and found significant increases in ED visits for multiple causes [19].

Here we seek to replicate and expand the evidence of heat-health effects in children across the US, leveraging a very large healthcare claims database with information on ED visits in children with health insurance living across the US. Specifically, we sought to characterize the association between warm-season temperatures and risk of ED visits for all causes and a series of specific causes among children aged <18 years with commercial health insurance. We additionally evaluated whether the observed associations varied by age, sex, and geographic region.

## 2. Materials and methods

### 2.1. Study population

We obtained de-identified medical claims records from 1 January 2016, to 31 December 2019, from the Optum Labs Data Warehouse, which includes medical and pharmacy claims, laboratory results, and enrollment records for commercially insured and Medicare Advantage enrollees [33]. The database contains longitudinal health information on enrollees representing a range of ages, ethnicities, and geographical regions. We identified claims for ED visits based on the International Classification of Diseases (ICD-10), revenue, Current Procedural Terminology, and place of service codes. For each claim, we then extracted information on the age, sex, and county of residence of the individual, as well as the admission date (defined as the date of service performed in the ED) and diagnosis codes. We limited our analysis to ED visits occurring among children (individuals aged <18 years). This study involved analysis of pre-existing, de-identified data and was approved by the Institutional Review Board of Boston University (IRB number: H-40274).

**Figure 1.**
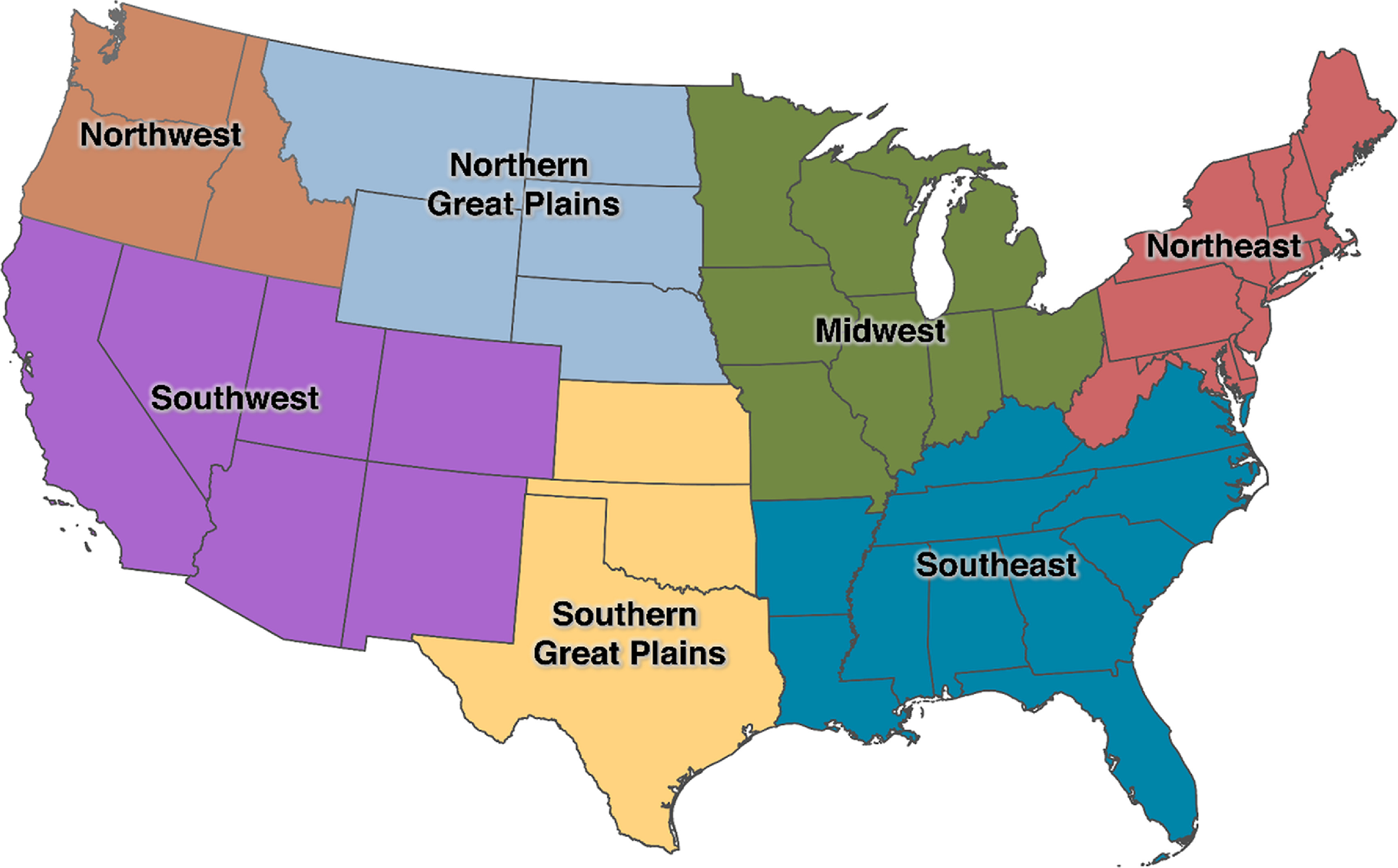
Climate regions in the United States. Locations of climate regions in the conterminous US based on the Fourth National Climate Assessment (NCA4).

In addition to ED visits for any cause (including accidental), we considered a range of specific causes for ED visits based on principal ICD diagnosis codes (table 1). Groupings are mutually exclusive, with the exception of asthma, bacterial enteritis, and suicidality/depression, which are included in more than one group. We additionally classified ED visits as heat-related based on any mention of relevant codes. We aggregated the daily number of ED visits in each county by age (0–5, 6–12, or 13–17 years), sex (male or female), and geographic region of the country as defined by the US Global Change Research Program’s Fourth National Climate Assessment (NCA4, figure 1) [34].

**Table 1.** Number of ED visits for any cause or specific causes among US children aged <18 with commercial health insurance, May thru September 2016–2019.

Health outcome	ICD-10^a^	Number of ED visits	% of total
All-cause	A00-Z99	1188 438	—
Infectious and parasitic diseases	A00-B99	48 472	4.08
Bacterial enteritis	A00-A09	7111	0.60
Blood and immune system disorders	D50-D89	4550	0.38
Endocrine, nutritional, metabolic diseases	E00-E85, E88-E89	4521	0.38
Mental, behavioral disorders	F00-F99	26 366	2.22
Nervous system diseases	G00-G99	16 309	1.37
Otitis media and externa	H60, H65-H67	48 245	4.06
Cardiovascular diseases	I00-I99	5699	0.48
Respiratory system diseases	J00-J99	157 927	13.29
Asthma	J45	21 754	1.83
Digestive system diseases	K00-K93	56 498	4.75
Skin and soft tissue infections	L00-L08	24 458	2.06
Other skin and soft tissue diseases	L09-L99	24 699	2.08
Musculoskeletal system diseases	M00-M99	42 181	3.55
Genitourinary system diseases	N00-N99	34 000	2.86
Perinatal conditions	P00-P96	9026	0.76
Other signs and symptoms	R00-R99	268 317	22.58
Suicidality and depression	R45.85-R45.87, R45.1, R45.4-R45.6, F32, F33	16 579	1.40
Injury and poisoning	S00-T66, T68-T88	467 762	39.36
Heat-related illness^b^	T67, E86, E87	25 362	2.13
External causes and other health factors	V01-Z99	25 835	2.17

^a^ Diagnosis codes as defined by the 10th revision of the International Statistical Classification of Diseases and Related Health Problems.

^b^ Based on principal or secondary diagnosis codes for heat-related illness, and principal diagnosis codes otherwise.

### 2.2. Ambient temperature

We estimated daily maximum ambient temperature (hereafter, simply ‘temperature’) and relative humidity using the Parameter-elevation Relationships on Independent Slopes (PRISM) dataset [35, 36]. The PRISM dataset is a publicly available gridded climate dataset that includes daily estimates of temperature, dew point, vapor pressure deficit, and precipitation with a horizontal grid spacing (spatial resolution) of approximately 4 km. To represent population exposure to temperature, we calculated a population-weighted average of daily maximum temperature for each day in each county, as previously described [37]. Briefly, we extracted individual temperature pixels from the PRISM dataset at the population centroids for each census tract and aggregated to the county level using a weighted average based on proportion of county population within each tract. To account for missing data, the weighting relied on the proportion of the population within each county with non-missing data: county-days with more than 20% missing data were excluded from the estimates (this affected <0.01% of county-days). We restricted this analysis to the months of May to September each year (henceforth referred to as ‘warm-season’ for brevity) and calculated percentiles of maximum temperature and relative humidity by day and county during the study period. Specifically, we defined a day of extreme heat as one where the daily maximum temperature exceeded a location-specific threshold based on the 95th percentile of the county-specific distribution of warm-season daily maximum temperatures during the study period.

### 2.3. Statistical approach

Using a case-crossover modeling framework, we estimated the relationship between county-specific daily maximum temperature percentile and all-cause and cause-specific ED visits among children during the warm seasons of 2016–2019. Under certain assumptions and conditions, the case-crossover analysis provides results that are similar to those of traditional time-series studies with Poisson regression [38–40]. In the case-crossover design, each participant serves as their own control, with inference based on the comparison of exposures over time within the same individual [41, 42]. This design has the advantage of controlling for all known and unknown individual- and county-level confounders that are time-invariant or vary relatively slowly over time, including, for example, age, sex, socioeconomic status, and population density [42]. We used a time-stratified approach to select control periods such that ambient temperature during the case period was compared to ambient temperature on other days within the same year, month, and day of the week as the case day [43]. This approach to selecting control periods serves to minimize confounding by seasonal and long-term time trends, as well as day of the week [43].

We used a conditional logistic regression model to estimate the association between daily maximum temperature percentile and the relative risk of ED visits. We applied a well-established distributed lag non-linear modeling framework to allow for both non-linear exposure-response functions and non-linear lag-response functions [44]. As in prior studies, we modeled exposure-response functions using a quadratic B-spline with one internal knot placed at the 50th percentile of the county-specific warm-season temperature distribution and modeled the lag-response function using a natural cubic B-spline with two knots placed at equal intervals on the log scale of lags up to five days [45]. We additionally adjusted for a natural spline function with 3° of freedom for daily mean relative humidity and federal holidays (as an indicator variable) in all models. We report odds ratios (OR) and 95% confidence intervals (CI) of ED visits associated with a day of extreme heat (defined as ambient temperature above the county-specific 95th percentile temperature distribution) versus a typical daily temperature (defined as the 50th percentile county-specific temperature) [44, 45].

We performed several sensitivity analyses to assess the robustness of our findings. First, we varied the key modeling parameters, including modeling exposure-response functions using a quadratic B-spline with two and three internal knots and modeling the lag-response function using a natural cubic B-spline with three knots placed at equal intervals on the log scale of lags up to five days. Second, to assess whether our results were robust to the choice of exposure metric, we repeated the main analyses using exposure based on county-specific percentiles of daily mean rather than daily maximum temperatures. Finally, to assess the impact of temperature alone, we repeated the main analyses without controlling for relative humidity.

### 2.4. Subgroup analyses

To examine potential differences in susceptibility, we evaluated whether the association between warm-season temperature and risk of ED visits varied across strata defined by age, sex, and NCA4 region, as defined above. We used the Wald test to assess whether the associations were homogeneous across strata [46]. All analyses were conducted using R version 4.0.2 with the ‘survival’ (version 3.1.12) and ‘dlnm’ (version 2.4.2) statistical packages [47, 48].

## 3. Results

### 3.1. Temperature and population

**Figure 2.**
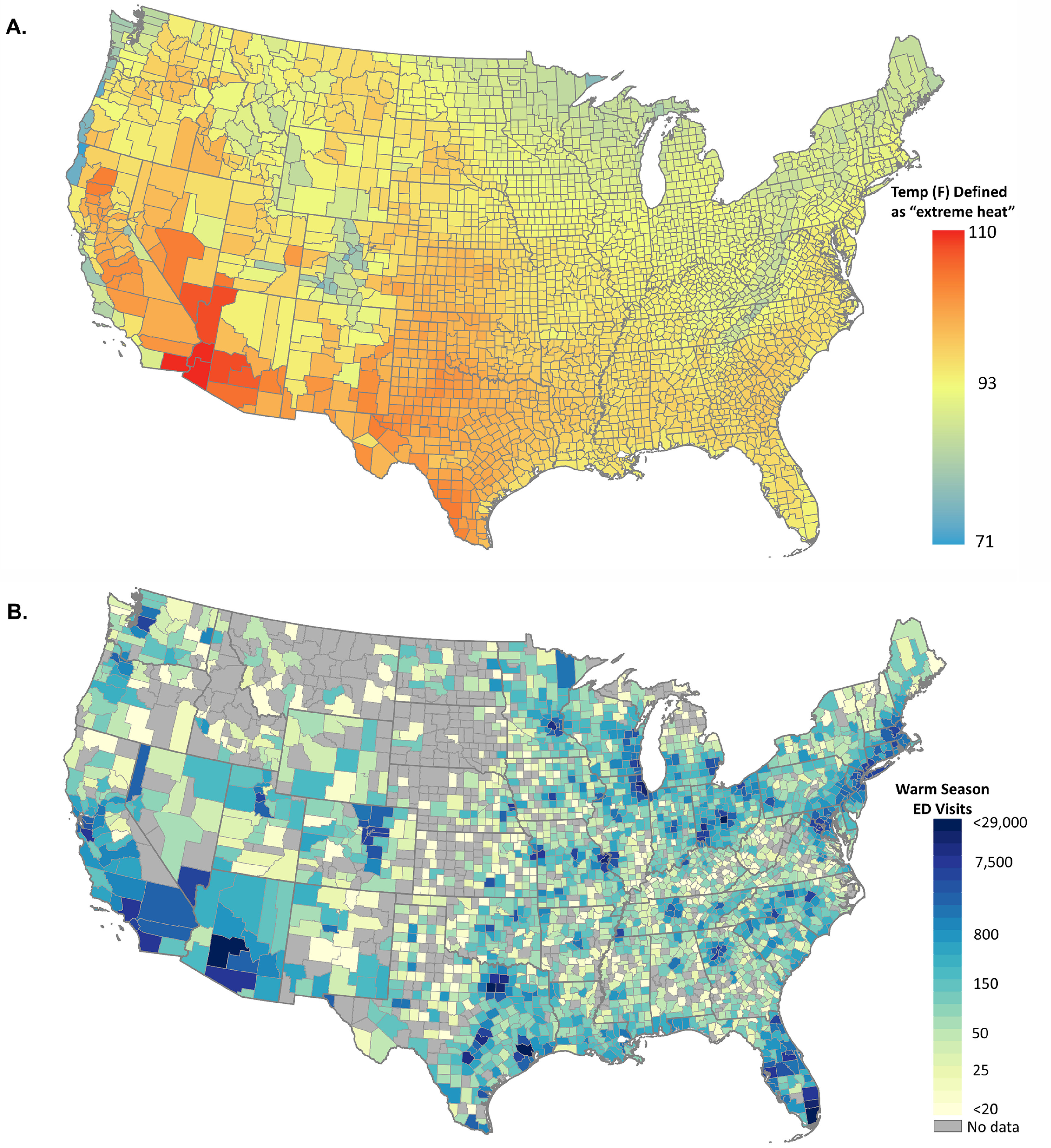
Maps showing the spatial distribution of the definition of days of extreme heat (A) and the number of ED visits included in the analyses (B). Figure (A) shows the warm- season (MJJAS) 95th percentile of daily maximum temperature (°F) observed at the same location from 2016 to 2019. Figure (B) shows the total number of warm-season ED visits per county among children aged <18 years of age with commercial health insurance during the same time period.

Warm-season daily maximum temperatures vary considerably across the US, with higher temperatures typically observed across the Southwest and Southeastern regions of the US (figure 2(A)). Our study population included approximately 4.7 million unique children with commercial health insurance on any given day, accounting for approximately 6.5% of the 2019 US resident population aged <18 years. We recorded approximately 1.2 million ED visits during the warm seasons between 2016 and 2019 among beneficiaries residing in 2489 of the 3109 counties in the contiguous US (figure 2(B)). Approximately 97% of the total US population <18 years lived in the counties in which we identified at least one ED visit in the Optum Labs administrative claims data. The most common causes of ED visits included injury and poisoning, non-specific signs and symptoms, and diseases of the respiratory system (table 1). ED visits specifically coded as ‘heat-related’ accounted for 2.6% of all ED visits.

### 3.2. Impact of warm-season temperatures

**Figure 3.**
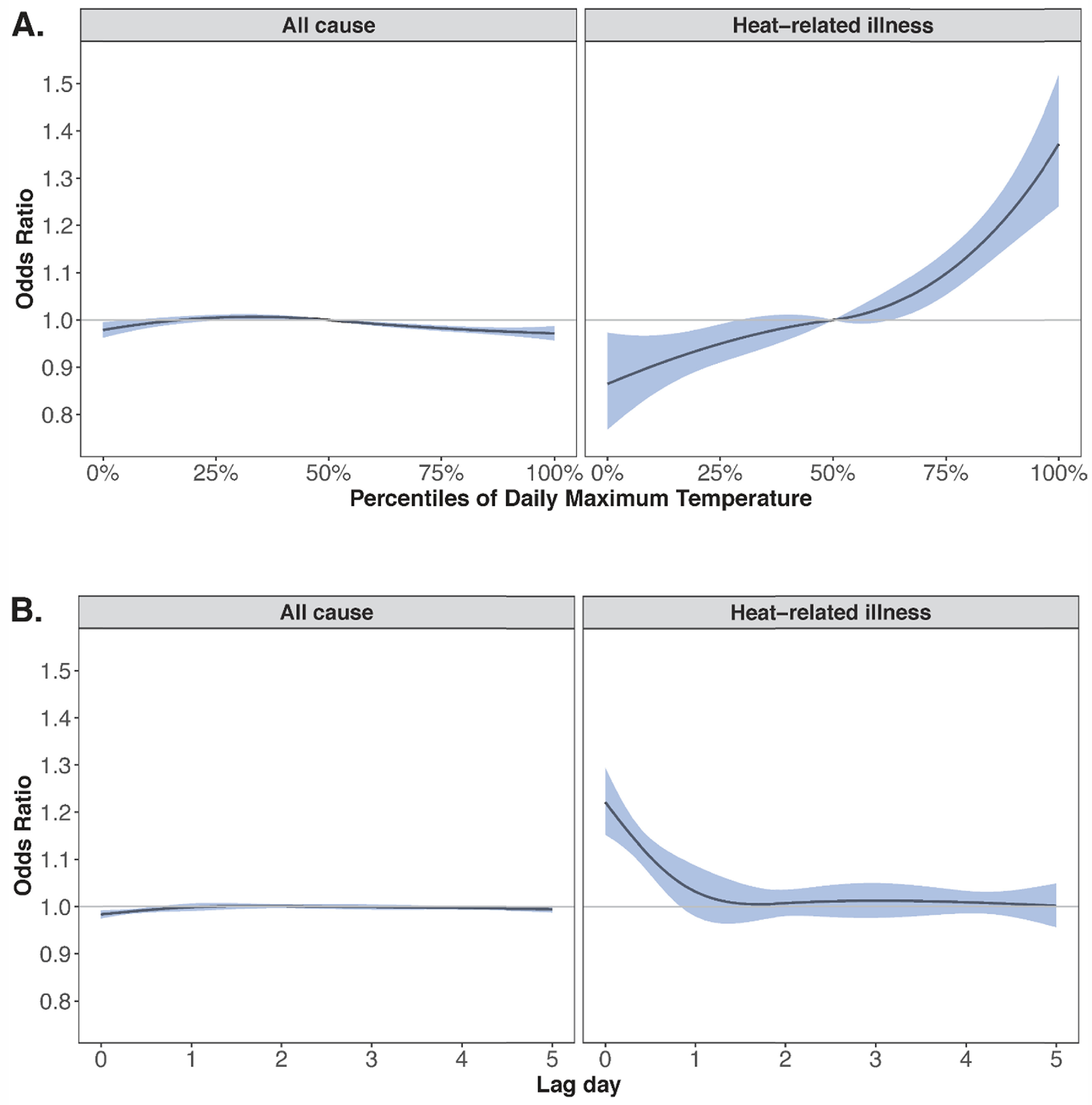
Association between daily maximum temperature and all-cause (left) and heat-related (right) ED visits. The top row of plots (A) depicts the non-linear association between percentiles of daily maximum temperature and the relative risk of ED visits, centered at the 50th percentile. The bottom row of plots (B) depicts the non-linear lag-response function for days at the 95th versus 50th percentile of daily maximum temperature.

Warm-season temperatures were not positively associated with higher rates of all-cause ED visits but were associated with higher rates of ED visits for heat-related illnesses (figure 3(A)). For example, a day of extreme heat was associated with a 30% (95% CI: 20%, 40%) higher rate of ED visits for heat-related illnesses versus the local median warm-season temperatures cumulatively over five days of lag. Exposure-response curves for all outcomes by temperature percentile are shown in supplemental figure 1. The increase in risk was highest on the same day (lag 0), with minimal evidence of lagged effects (figure 3(B) and supplemental figure 2).

**Figure 4.**
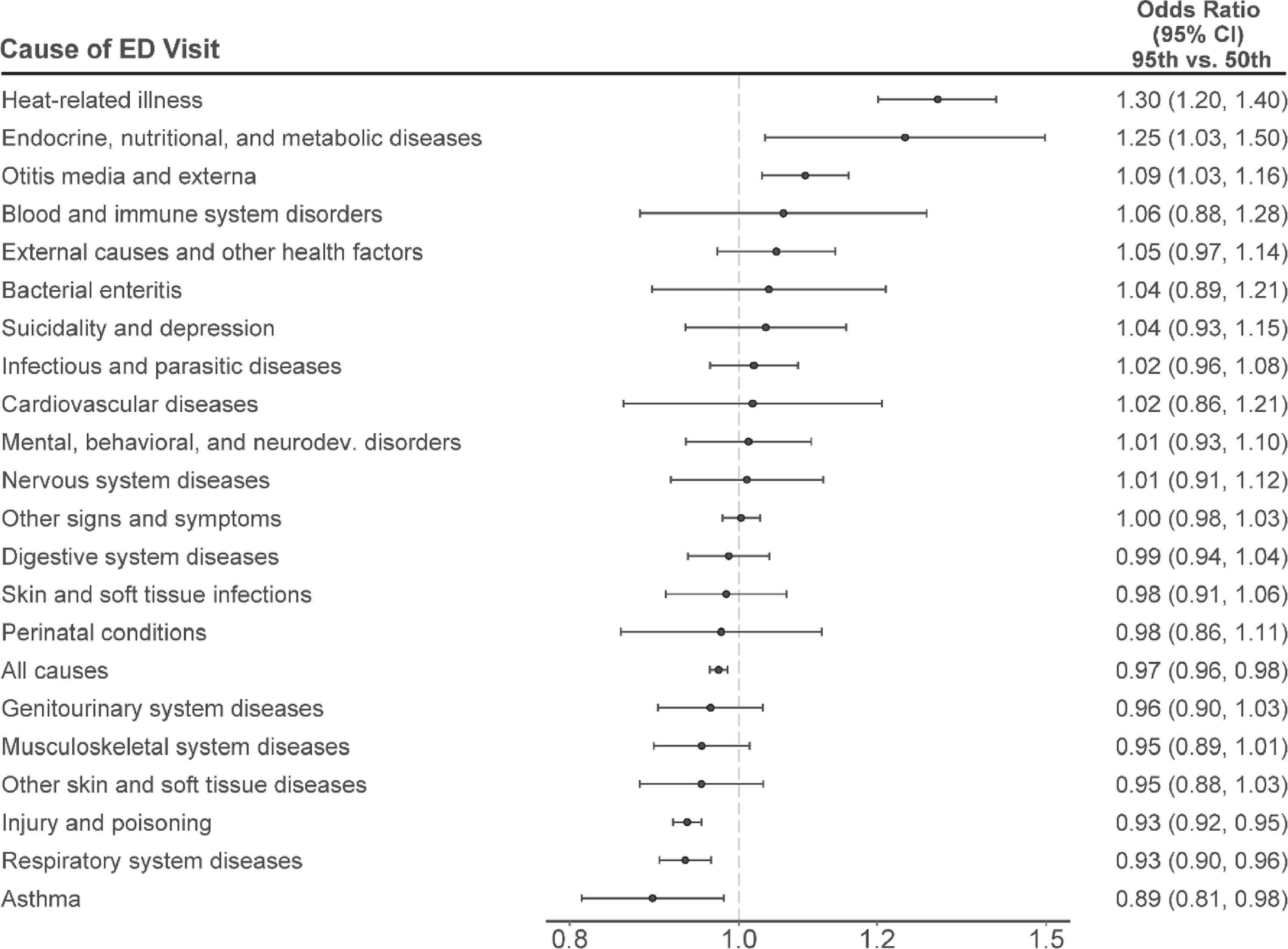
Odds ratios and 95% confidence intervals of the association between temperature and cause-specific ED visits, expressed as a comparison between the 95th to the 50th percentile of the daily maximum temperature.

Warm-season temperatures were also associated with higher rates of ED visits for endocrine, nutritional, and metabolic diseases and otitis media and externa (figure 4). Specifically, a day of extreme heat versus the local median warm-season temperature was associated with a 25% (95% CI: 3%, 50%) higher rate of ED visits for endocrine, nutritional, and metabolic diseases and a 9% (95% CI: 3%, 16%) higher rate for otitis media and externa. We did not observe evidence of a positive association between extreme heat and rates of ED visits for any other specific cause. On the other hand, days of extreme heat were associated with lower rates of ED visits for diseases of the respiratory system, injury and poisoning, and asthma. For ease of comparison to other studies, results were recalculated comparing the 95th percentile to both the 1st percentile and mean of warm-season maximum temperatures (supplemental figures 3 and 4). Results were not materially different in sensitivity analyses in which we varied model parameters (supplemental figure 5).

### 3.3. Stratified analyses

**Figure 5.**
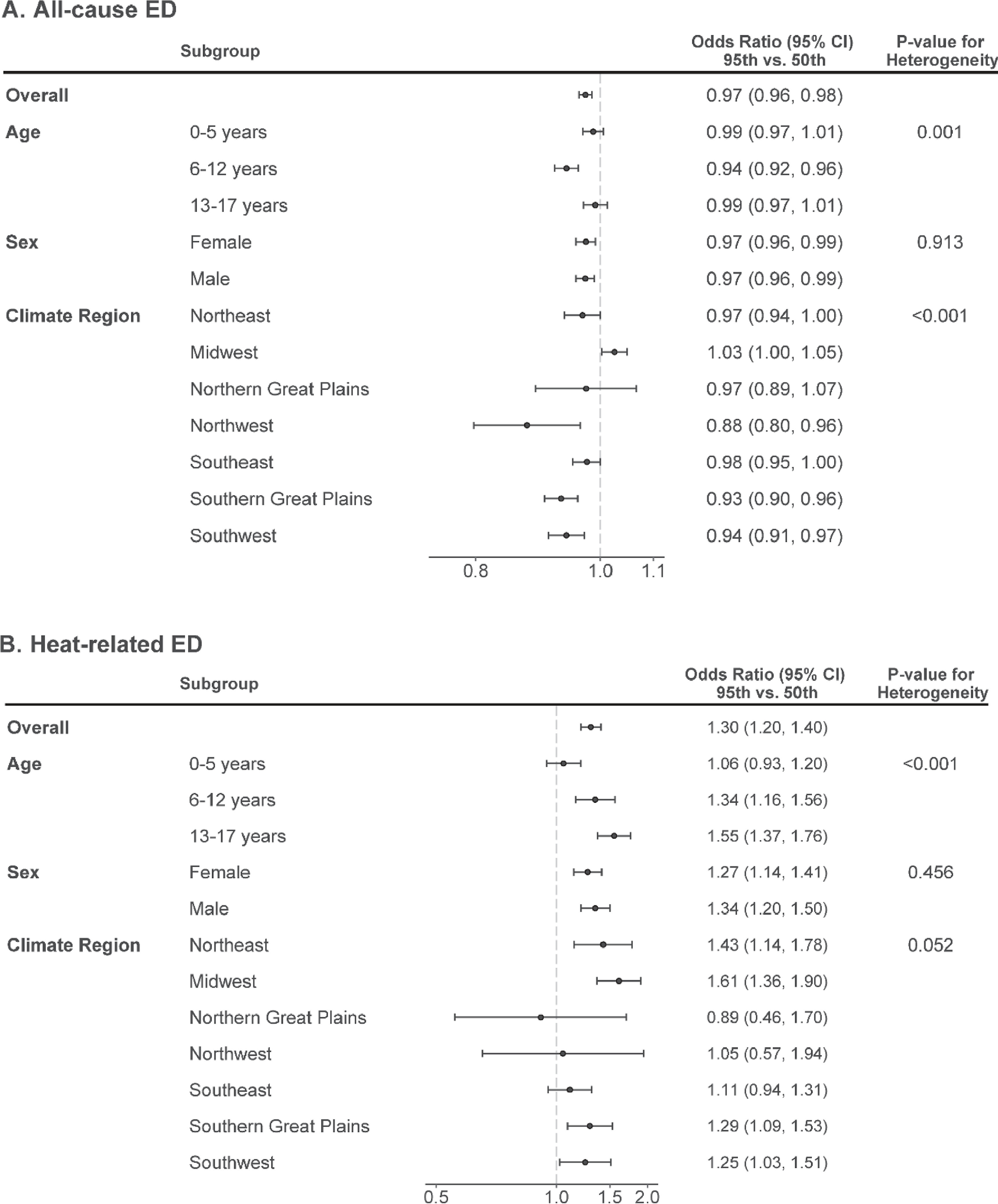
Odds ratios and 95% confidence intervals of the association between temperature and all-cause (A) or heat-related (B) ED visits, overall and within subgroups defined by age, sex, or climate region.

We assessed whether the associations between warm-season temperature and ED visits for all-cause and heat-related illness varied across subgroups defined by age, sex, and region (figures 5(A) and (B)). Warm-season temperatures were not associated with higher rates of all-cause ED visits in any subgroup (age, sex, and climate region), except in the Midwest. Indeed, in several subgroups, warm-season temperatures were associated with lower rates of all-cause ED visits.

On the other hand, the association between temperature and ED visits for heat-related illnesses varied across strata defined by age, with increasing relative risks across strata of increasing age. For example, a day of extreme heat was associated with relative risks of heat-related ED visits of 1.06 (95% CI: 0.93, 1.20), 1.34 (1.16, 1.56), and 1.55 (1.37, 1.76) for children aged 0–5 years, 6–12 years, and 13–17 years, respectively. The association between temperature and heat-related ED visits were not materially different across subgroups defined by sex. Associations with heat-related ED visits varied somewhat across regions of the country, with stronger associations observed in the Midwest and Northeast. However, estimates in some regions were imprecise and overall heterogeneity was modest.

## 4. Discussion

Despite ample evidence that extreme heat poses a significant public health risk in adults, relatively few studies have evaluated the impact of warm-season temperatures on children. We leveraged a large, national dataset of healthcare utilization claims to quantify the association between warm-season temperatures and rates of ED visits for any cause and for specific causes among a large sample of children and adolescents <18 years old with commercial health insurance. We found that higher temperatures were not associated with higher rates of ED visits overall but were associated with higher rates of ED visits for heat-related illness; endocrine, nutritional, and metabolic diseases; and otitis media and externa.

The limited number of prior studies available in children collectively suggests that extreme heat is associated with higher rates of ED visits for a range of causes [15, 26, 27, 30, 49]. For example, Bernstein *et al*, used a national dataset of 3.8 million ED visits from 47 children’s hospitals across the US, regardless of health insurance coverage, and found an 83% increase in heat-related ED visits associated with days of extreme heat (comparing the 95th versus 1st percentiles of location-specific daily maximum temperature). Similar positive associations between warm-season temperatures and healthcare utilization have been documented among children <15 years of age in London [15].

Few studies have been published regarding the effects of high temperatures on otitis media and externa, as well as endocrine, nutritional, and metabolic diseases. For example, Bernstein *et al* estimated a 30% increase in otitis media and externa ED visits associated with days of extreme heat but did not observe a significant relationship between temperature and endocrine, nutritional, and metabolic disease in children [19]. The authors of a study focused on relative humidity also investigated the potential effects of temperature on otitis media-related ED visits and failed to find a statistically significant association [50]. Finally, Xu *et al,* investigated a relationship between high temperatures and ED visits for endocrine, nutritional and metabolic diseases in Australian children and found statistically significant associations for cumulative lags 0–21 days [49].

Our finding that warm-season temperatures were not associated with higher rates of all-cause ED visits stands in contrast with most prior studies [19, 26, 31, 32]. For example, Bernstein *et al* found a 17% increase in rates of ED visits for any cause on days of extreme heat (comparing the 95th versus 1st percentile of daily maximum temperatures) [19]. Similarly, among children aged <17 years of age in Southwestern Ontario, Wilk *et al* found 22% higher rates of all-cause ED visits comparing 99th percentile maximum temperature with a reference absolute temperature of 21 °C [31]. Knowlton *et al* found higher rates of all-cause ED visits in California for two age categories that included children, with a 5% increase in ED visits for ages 0–4 years and a 3% increase for individuals 5–64 years of age when comparing a heatwave period to a non-heatwave reference period [26]. Davis *et al* investigated the impact of heat waves in Virginia and found higher rates of ED visits for all causes in children 1–4 years and 10–19 years (20% and 13%, respectively) [32]. Of note, our definition of all-cause ED visits included visits for external causes such as injury and poisoning, whereas some other studies have excluded external causes from their definition of ‘all-cause’. Variation in the definition of the outcome may account for some of the heterogeneity seen across studies.

On the other hand, we observed a negative association between heat and respiratory illness, a finding that warrants further investigation. Multiple studies have found positive associations between temperature and respiratory illness [51–54]. For example, in Tchidjou *et al*, maximum temperature was positively associated with hospital admissions for all respiratory diagnoses in children 0–17 years [51]. Other studies have found either no association or a negative association; for example, Grech *et al* found negative associations between temperature and asthma for children 0–14 years old, and Loh *et al* similarly found a protective effect of temperature in children 0–5 years [52, 54]. One potential explanation for these discrepant results is that higher temperatures have a differential impact on individual behavior in different locations or populations. For example, although many of the design and analysis details were similar, the results of this study (limited to children with commercial health insurance seen at any ED in the US) are somewhat different than those of Bernstein *et al* (limited to US children visiting one of 47 children’s hospitals, regardless of insurance status). This reinforces the notion that vulnerability to heat can vary substantially across subgroups of the population.

Children may respond differently to heat than adults. In a previous study, we utilized the same source population and similar methodology to look at the impact of warm-season temperatures on adults [5]. Comparing the results of these two studies reveals both similarities and differences in the impacts of heat. While both studies indicated statistically significant positive associations between temperature and heat-related illness, the excess relative risk was slightly higher in adults versus children (66% vs. 50%, respectively, when comparing 95th vs 1st percentile of temperature). On the other hand, in children, heat was not associated with all-cause ED visits and negatively associated with ED visits for mental health disorders, while in adults heat was positively associated with both endpoints.

Our findings suggest that risk for heat-related illness may depend on the age of the child, with heat being more weakly associated with ED visits in younger children (0–4 year) versus older children (5–12 year) and adolescents (13–17 year). Older children and adolescents may tend to spend more time outdoors with less adult supervision, potentially contributing to higher or prolonged exposures to heat and/or fewer protective actions such as seeking shade or staying hydrated. Vulnerability to heat may also vary across geographic regions based on typical climate, and a host of related factors such as distribution of green space, severity of urban heat islands, and availability of residential air conditioning. We did find some evidence of variability in the impacts of temperature on the risk of heat-related ED visits, but wide confidence intervals in some regions make it hard to draw many conclusions from these findings.

The data used in this study were obtained from children and adolescents with commercial health insurance, excluding children with public health insurance or no health insurance who may be more vulnerable to the health impacts of heat. Thus, our results may not be generalizable to children without commercial health insurance. The propensity for seeking care in an ED may also vary by insurance status, with children on public insurance or no insurance being more likely to seek care in the ED [55]. Other potential limitations of this study include exposure misclassification due to aggregation to the county level, lack of data on individual time-activity patterns (e.g. time spent outside), and lack of data on access to air-conditioned spaces either at home or at school. Moreover, lack of information on individual level socio-economic or other characteristics limited our ability to investigate differences in vulnerability across subgroups of the population.

Nonetheless, the findings from this comprehensive study in a very large population across the US provide further evidence that heat poses an important risk to children and adolescents, as well as adults. The differences between the findings of this and some prior studies also highlight the need for additional research to better understand how vulnerability to heat in children varies by individual and neighborhood-level characteristics as well as the pathophysiologic processes underlying the observed associations. More importantly, additional research on the effectiveness of existing and new interventions is needed to reduce the impacts of heat on children’s health. The need for this research is urgent as extreme heat events are expected to increase in frequency and severity with continued climate change [56].

## 5. Conclusions

In summary, exposure to high ambient temperatures can be detrimental to children’s health. In a large population of children and adolescents with commercial health insurance, we found that days of extreme heat were associated with higher rates of healthcare utilization for heat-related illnesses; endocrine, nutritional and metabolic diseases; and otitis media and externa. For heat-related illnesses, children above the age of four may experience a greater risk of an ED event after exposure to high temperatures. Additional research to better understand the health impacts of heat on children, and how to reduce these effects, is essential.

## Data Availability

The exposure data that support the findings of this study are available upon reasonable request from the authors. Health data used in this study are only available by contacting Optum Labs to obtain access to the insurance claims data.
