# Supplementary material for: Warm-Season Temperatures and Emergency Department Visits among Children with Health Insurance
Source: Environ Res Health. Author manuscript; Available in PMC 2023 Mar 1. (PMC9623446; doi:10.1088/2752-5309/ac78fa)

**Supplemental Data**

**Supplemental Figure 1.** Cumulative exposure-response curves for all cause and cause-specific ED events by maximum temperature percentile (95^th^ vs 50^th^ percentile), May thru September 2016-2019.**
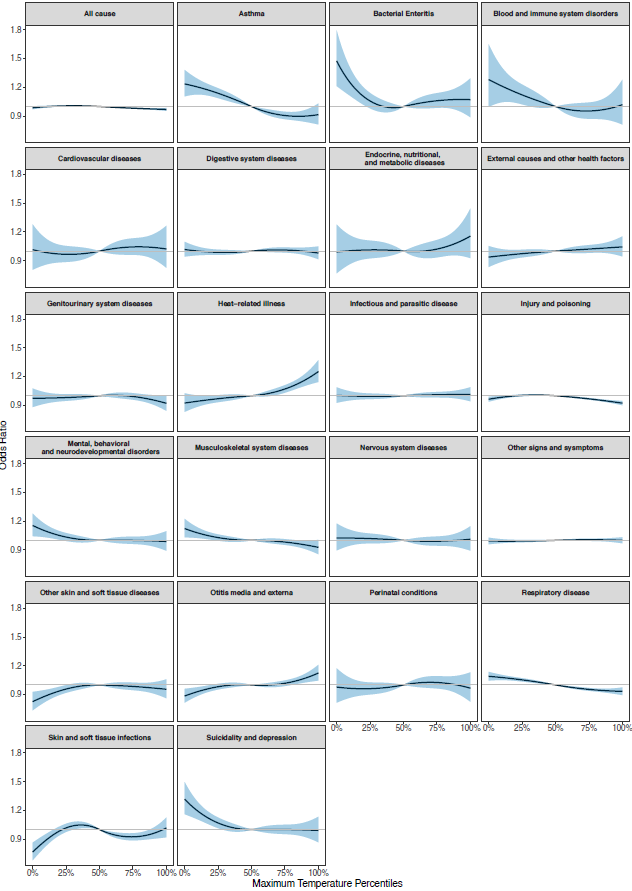
**

**Supplemental Figure 2.** All cause and cause-specific ED odds ratios and 95% confidence interval for 95^th^ vs. 50^th^ percentile temperature over lag 0-5 days May to September of 2016-2019.
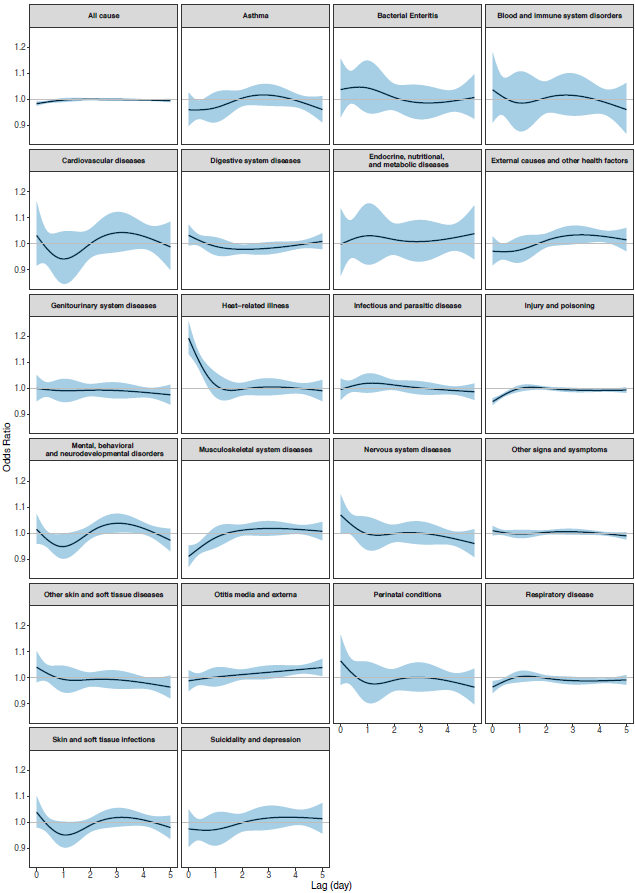


**Supplemental Figure 3.** All cause and cause-specific ED odds ratios and 95% confidence interval for 95^th^ vs. 1^st^ percentile temperature May to September of 2016-2019.


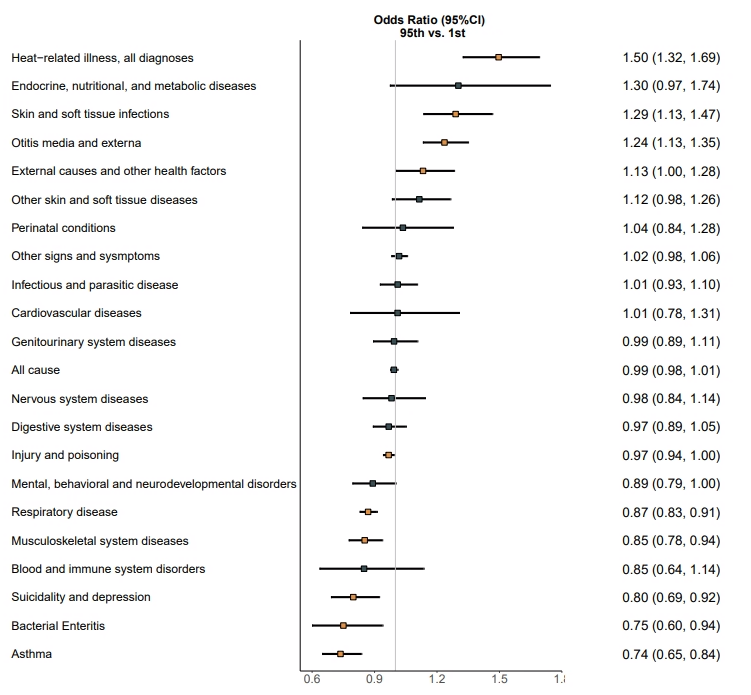


**Supplemental Figure 4.** All cause and cause-specific ED odds ratios and 95% confidence interval for per 10° C increase of daily mean temperature over lag 0-5 May to September of 2016-2019.


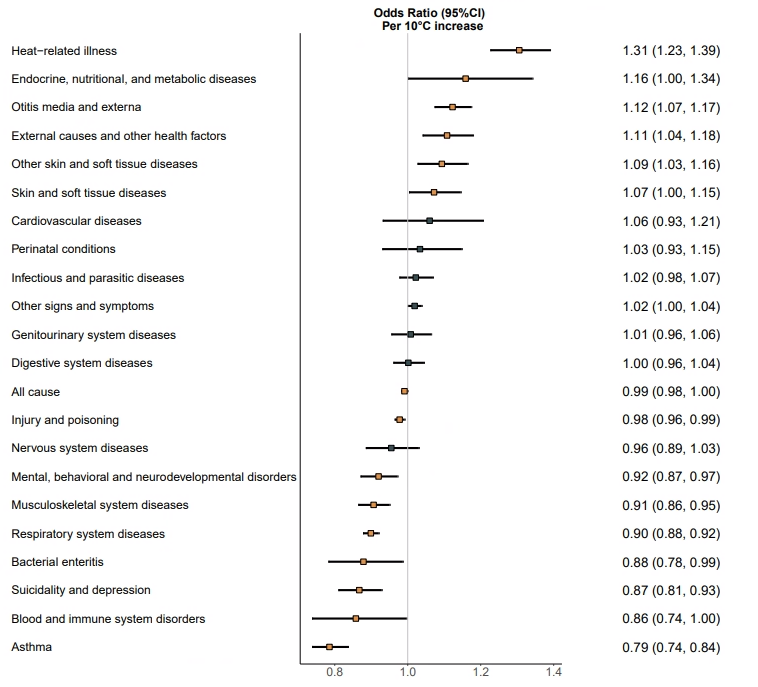


**Supplemental Figure 5.** Multiple sensitivity analyses adjusting for location and number of knots for temperature, additional knot for lagged analysis, and exclusion of relative humidity for May-September 2016-2019. A) Cumulative exposure-response curves for all cause and cause-specific ED events by maximum temperature percentile, B) All cause and cause-specific ED odds ratios and 95% confidence interval and 95^th^ percentile temperature over lag 0-5 days.


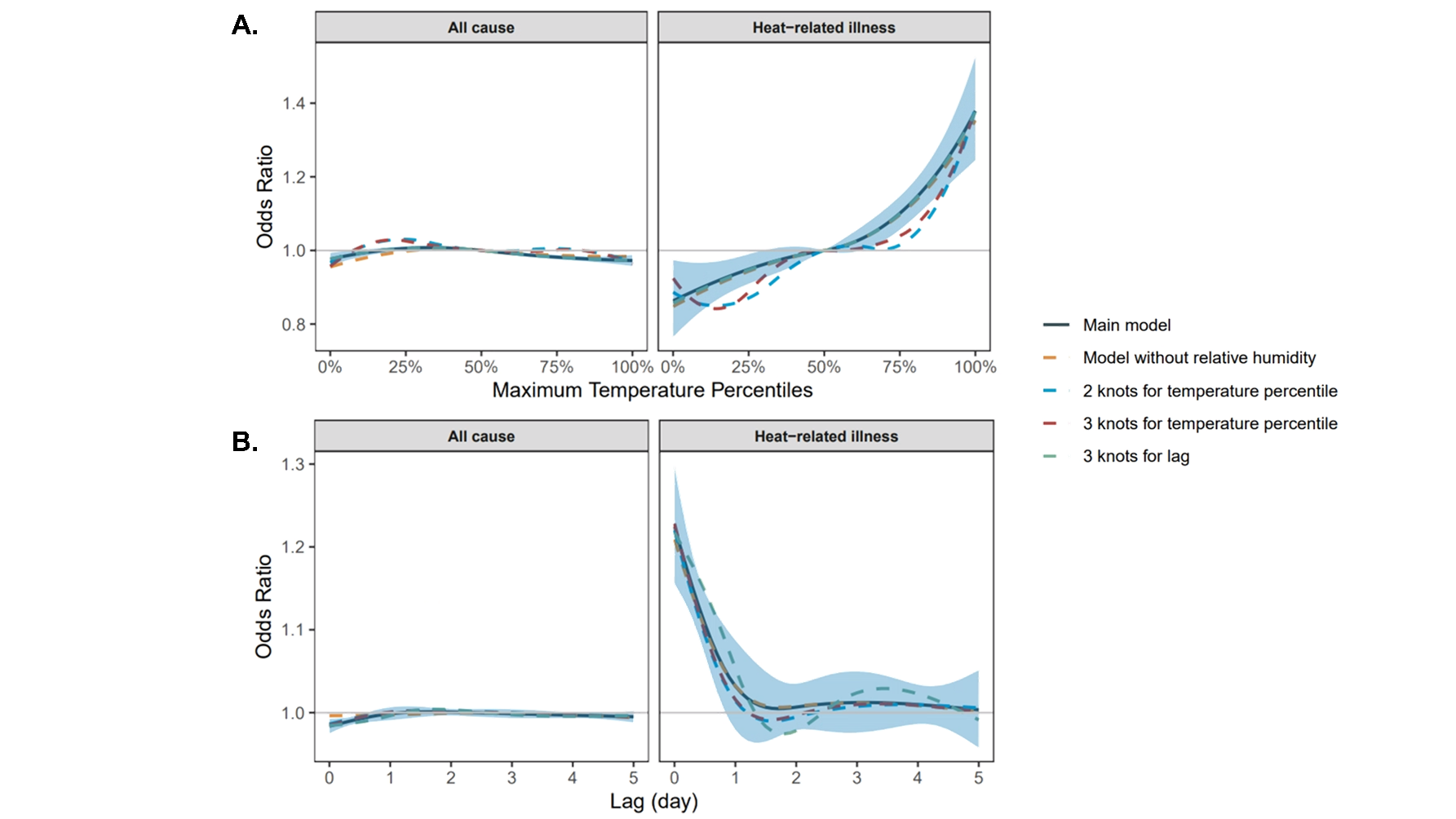

Supplement: Supplemental [file NIHMS1826261-supplement-Supplemental.docx]
